# Chinese College Students' Stress and Anxiety Levels Under COVID-19

**DOI:** 10.3389/fpsyt.2021.615390

**Published:** 2021-06-10

**Authors:** Huali Zhan, Chunmei Zheng, Xianqin Zhang, Meng Yang, Lin Zhang, Xu Jia

**Affiliations:** ^1^Department of Humanities and Social Sciences, Zhejiang Industry Polytechnic College, Shaoxing, China; ^2^Department of Psychiatry, The Seventh People's Hospital of Shaoxing, Shaoxing, China; ^3^School of Basic Medical Sciences, Chengdu Medical College, Chengdu, China; ^4^Non-coding RNA and Drug Discovery Key Laboratory of Sichuan Province, Chengdu Medical College, Chengdu, China; ^5^Department of Pharmacy, Shaoxing People's Hospital, Shaoxing Hospital, Zhejiang University School of Medicine, Shaoxing, China

**Keywords:** COVID-19, returning to school, college student, anxiety, stress

## Abstract

The outbreak of COVID-19 at the end of 2019 has had a significant impact on people. While attention is paid to the immense physical harm it has caused, the psychological impact should not be underestimated. The main purpose of this study was to explore the stress, anxiety, and depression levels of different groups of college students during the COVID-19 pandemic. We conducted an online questionnaire survey of college students by using the Perceived Stress Scale (PSS-10), the Patient Health Questionnaire-9 items (PHQ-9), and the Self-rating Anxiety Scale (SAS). A total of 1,586 questionnaires were collected and analyzed in R language. The results showed that students with moderate to severe stress (PSS-10 ≥ 14) accounted for 67.50%; the detection rate of depression (PHQ-9 ≥ 5) reached 43.77%; and 20.60% of students had anxiety (SAS standard score ≥ 50). There were significant differences in PSS-10/SAS among different genders, majors, whether returning to school or not, and those with different psychological experiences (negative or positive, *P* < 0.05). It is notable that the median of female, medical student, non-resumption of schooling, and negative experience was higher than that of positive experience (*P* < 0.05). The results of principal component analysis showed that there were significant differences in PHQ-9, PSS-10, and SAS between the resumption of schooling group and the non-resumption group. Therefore, it is inferred that the stress and anxiety level of college students during the COVID-19 pandemic is generally high, especially for those who have not yet resumed school. Long-term negative emotions can easily lead to serious mental diseases such as cognitive impairment. Education departments should attach great importance to the mental health of college students, and it is necessary to provide precise psychological interventions for groups experiencing greater pressure levels and marked anxiety and depression.

## Introduction

In December 2019 a novel coronavirus appeared, mainly causing a new coronavirus pneumonia, which the World Health Organization (WHO) named Coronavirus Disease 2019 (COVID-19). COVID-19 is highly contagious and can be transmitted directly from person-to-person through respiratory droplets, contact, excretion, among others (1). On January 31, 2020, the WHO called COVID-19 an “emergent public health event of international concern.” The rapid spread, high infectivity, risk of infection and death, strict isolation measures, and other influencing factors have had great psychological impact on most people, especially on the mental health of college students who have extensive interpersonal communication and have heavy learning tasks.

A recent study showed that COVID-19 has led to a sharp increase in the prevalence of anxiety and depression among ordinary adults in China (2). College students also experience anxiety and depression in varying degrees (3). The group of college students is a huge group of collective life. In order to stop the spread of the pandemic to college campuses, the start of the 2020 spring semester was delayed and students conducted online learning at home (4). They went out less and lacked interpersonal communication, creating the potential for psychological stress, anxiety, and even depression (5) which could eventually lead to long-term physical and mental illness. With the strict regulation of relevant government departments and the concerted efforts of people throughout the country, the pandemic situation has been controlled in China, with phased results. In line with the development of the local pandemic situation, colleges and universities reopened in an orderly manner. College students who have just returned to school have experienced stress, anxiety, and depression due to the sudden change of learning style and a return to collective life with roommates from different places. A study showed that the mental damage caused by this sudden global public health incident may turn into a long-term health problem (6). Therefore, education departments, colleges, and universities should pay attention to the psychological status of college students during the pandemic and take corresponding measures to guide them.

At present, while there are studies on the mental health of college students during the COVID-19 pandemic, no investigation has been done on the stress and emotion experienced by college students before and after the resumption of school in the later stages of the pandemic. In this study, a cross-sectional survey was conducted to explore the psychological stress and emotional problems of such college students. We evaluated the psychological influence of psychological stress, anxiety, and depression among college students, in order to facilitation the provision by education departments, colleges, and universities of more accurate psychological intervention.

## Materials and Methods

### Research Objects and Sampling

In this study, WeChat was used to distribute questionnaires online. A cross-sectional survey was conducted by random sampling from May 9 to May 22, 2020, and a total of 1,586 questionnaires were collected for data analysis. All respondents participated voluntarily.

### Research Methods

The Perceived Stress Scale (PSS), Patient Health Questionnaire-9 items (PHQ-9), Self-Rating Anxiety Scale (SAS), and general situation questionnaire were used as standardized measurement tools to collect social measurement data.

The PSS-10 scale was used to assess the stress intensity related to the respondents' living conditions over recent months. It contained 10 items, and each item was rated on a 5-point Likert scale (0 for never, 1 for occasionally, 2 for sometimes, 3 for often, and 4 for always), with a total score of 0 to 40. The higher the score, the higher the perceived stress level (0–13 indicated a low-pressure level, 14–19 medium pressure level, and >19 high-pressure level). The Chinese version of PSS-10 has high credibility (Cronbach's α = 0.91) (7).

The PHQ-9 plays a role in auxiliary diagnosis in depressive disorder, but can also be used to evaluate the severity of the disease. The scale had a total of 9 items, with a 4-level rating model (0 = none, 1 = Less than half the time, 2 = More than half the time, and 3 = almost every day). The total score is between 0 and 27, and the frequency of symptoms in the last 2 weeks are evaluated (8). When the total score reaches 7, it indicates that there may be clinical depression. A large number of clinical studies have shown that PHQ-9 is a self-rating scale with good reliability and validity for teenagers (Cronbach's α = 0.85) (9, 10).

The total gross score was obtained by adding up the scores of 20 items and a 4-point rating scale of 1–4 was used in SAS. The gross score was multiplied by 1.25 as the SAS standard score. The higher the score, the worse the anxiety symptom was. The scale has been widely used in China, and it has been proved to have high reliability and validity (Cronbach's α = 0.897)(11).

Finally, a conclusion was obtained based on the relevant data analysis of different grades, different majors, whether returning to school, and different psychological experiences. In addition, this study was approved by the Academic Ethics Committee of Shaoxing People's Hospital (Ethics Clearance No. 121). Electronic informed consents were obtained online. All participants were informed that they could withdraw from the study at any time.

### Statistical Methods

R Language (3.6.0) was used for data analysis. Dplyr package was used for data processing, ggstatplot package was used for visualization of comparative data between two samples, factoextra and ggplot2 packages were used for principal component analysis and visualization. The student *t*-test and Pearson correlation coefficient were used for the correlation between variables.

## Results

### Sample Characteristics

Table 1 shows the demographic characteristics and health status of all participants. Among 1,586 students who participated in the survey, graduates and non-graduates accounted for 3.13 and 96.87%, respectively. Males accounted for 36.32% and females accounted for 63.68%. Nearly three-fifths (59.52%) were medical majors and non-medical majors accounted for 40.48%. Students who had resumed their studies accounted for 21.69%. The news that school would be resuming had a certain psychological impact on college students, of which positive psychological effects accounted for 70.81% and negative psychological effects accounted for 29.19%.

**Table 1:** Demographic characteristics and health status of all participants.

	**Female**	**Male**	***p*-value**
***N***	1010	576	
**Grade (%)**			
Graduation Year	39 (3.9)	9 (1.6)	0.016
Non-graduate Year	971 (96.1)	567 (98.4)	
Medical profession (%)			
Yes	674 (66.7)	270 (46.9)	<0.001
No	336 (33.3)	306 (53.1)	
**Resuming school (%)**			
Yes	126 (12.5)	218 (37.8)	<0.001
No	884 (87.5)	358 (62.2)	
**Employment impact (%)**			
No	88 (8.7)	88 (15.3)	<0.001
Slight	486 (48.1)	268 (46.5)	
Significant	436 (43.2)	220 (38.2)	
**Psychological feeling (%)**			
Positive	669 (66.2)	454 (78.8)	<0.001
Negative	341 (33.8)	122 (21.2)	
**Adaptation (%)**			
General	593 (58.7)	324 (56.2)	<0.001
Not very	249 (24.7)	85 (14.8)	
Very	130 (12.9)	144 (25.0)	
Very not	38 (3.8)	23 (4.0)	
**PSS-10, mean (SD)**	16.52 (5.80)	15.25 (6.24)	<0.001
**PHQ-9, mean (SD)**	5.28 (5.51)	4.97 (5.52)	0.279
**SAS standard score, mean (SD)**	42.70 (9.95)	41.55 (10.41)	0.029

### Analysis of the Evaluation Results of Each Scale

#### Evaluation Results of PSS-10, PHQ-9, and SAS

The stress levels of the 1,586 college students surveyed were found to be either low, medium, or high accounting for 32.5, 41.33, and 26.17%, respectively. The detection rate of depression (PHQ-9 ≥ 5) was 43.77%, and the detection rate of anxiety symptoms (SAS standard score ≥ 50) was 20.60%.

#### Schematic Diagram of the Distribution of PSS-10, PHQ-9, and SAS in Different Groups of College Students

**Figure 1:**
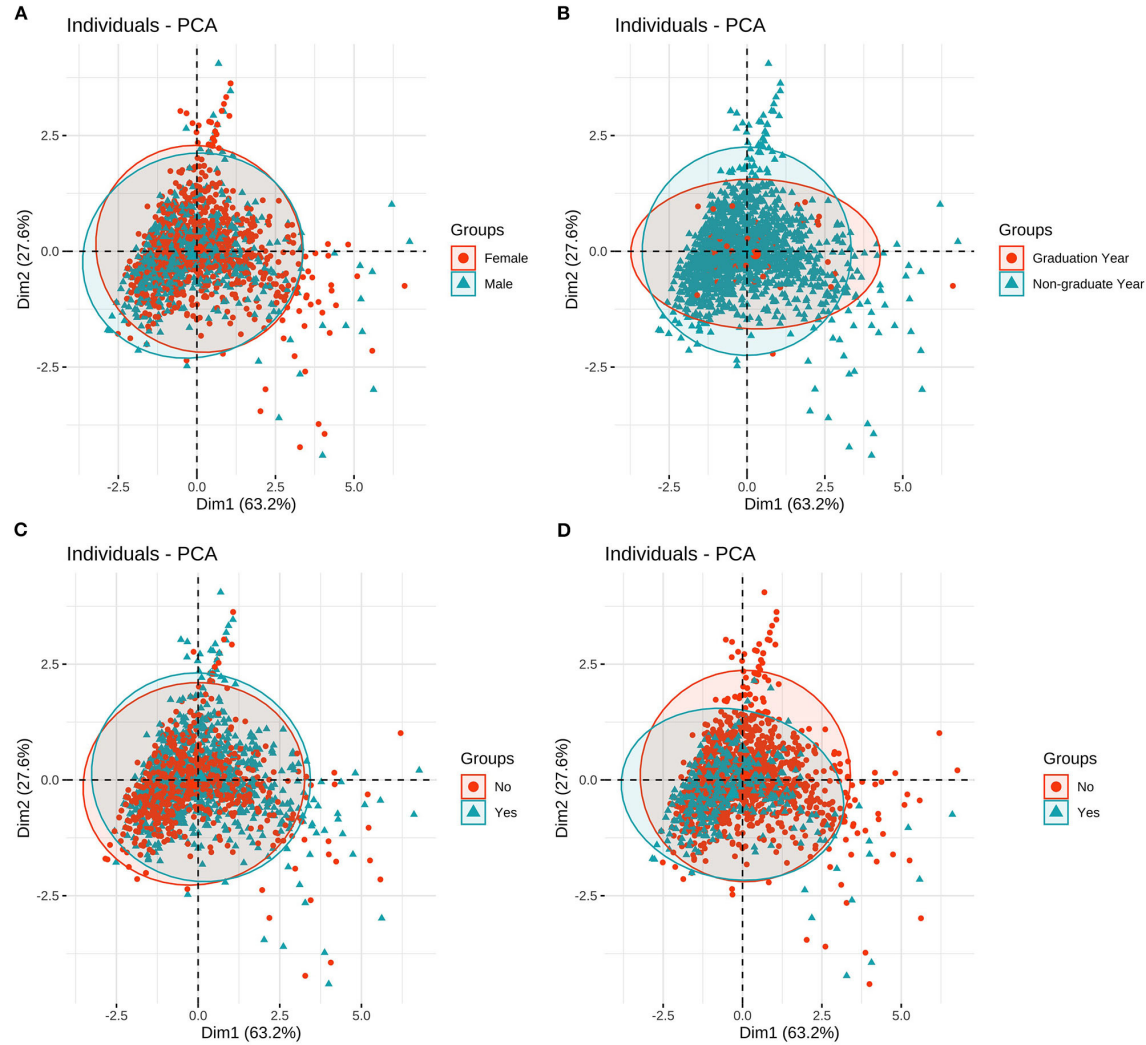
Principal component analysis of different groups. **(A)** Principal component analysis of different genders; **(B)** Principal component analysis of different grades; **(C)** Principal component analysis of different majors; **(D)** Principal component analysis of resuming-school or not.

**Figure 2:**
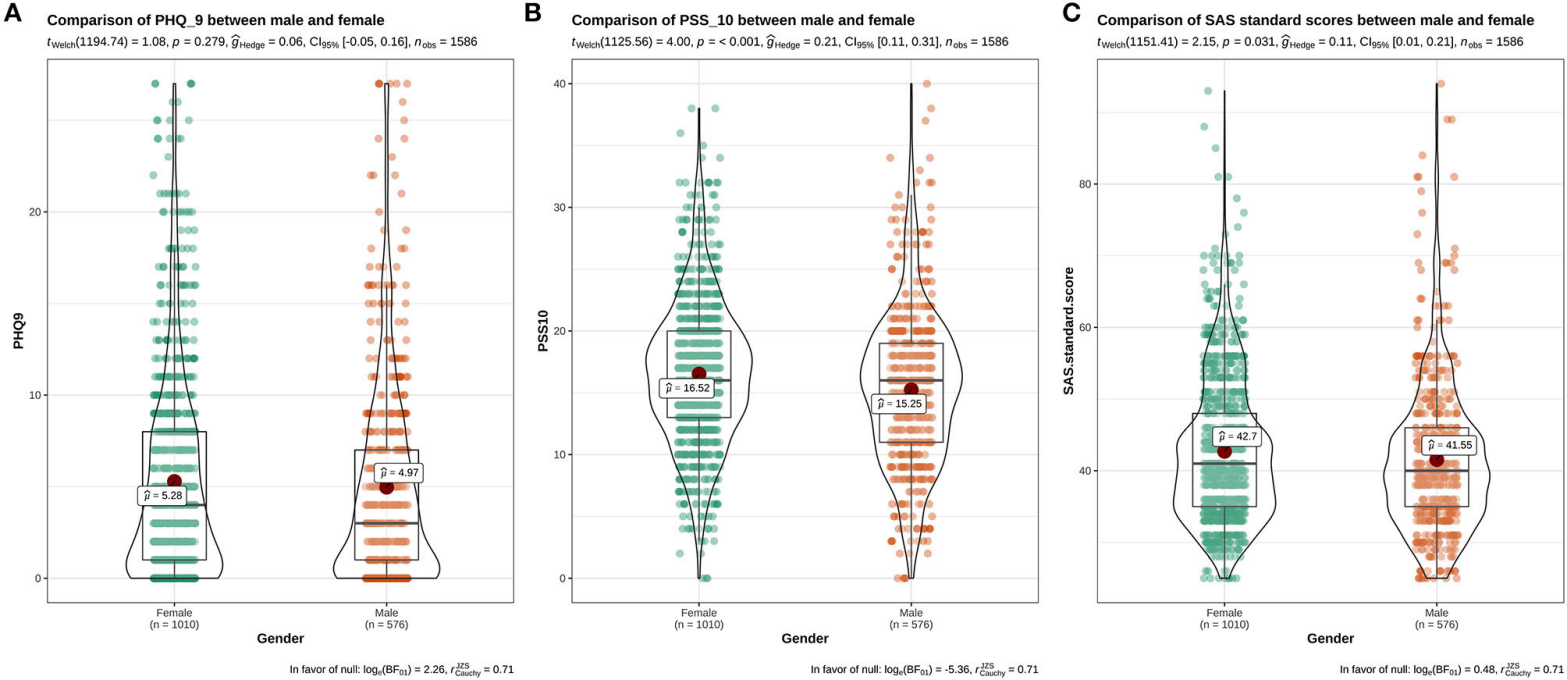
The distribution maps of PHQ-9, PSS-10, and SAS scores of different genders. **(A)** Distribution of PHQ-9 scores in different genders; **(B)** Distribution of PSS-10 scores in different genders; **(C)** Distribution of SAS scores in different genders.

**Figure 3:**
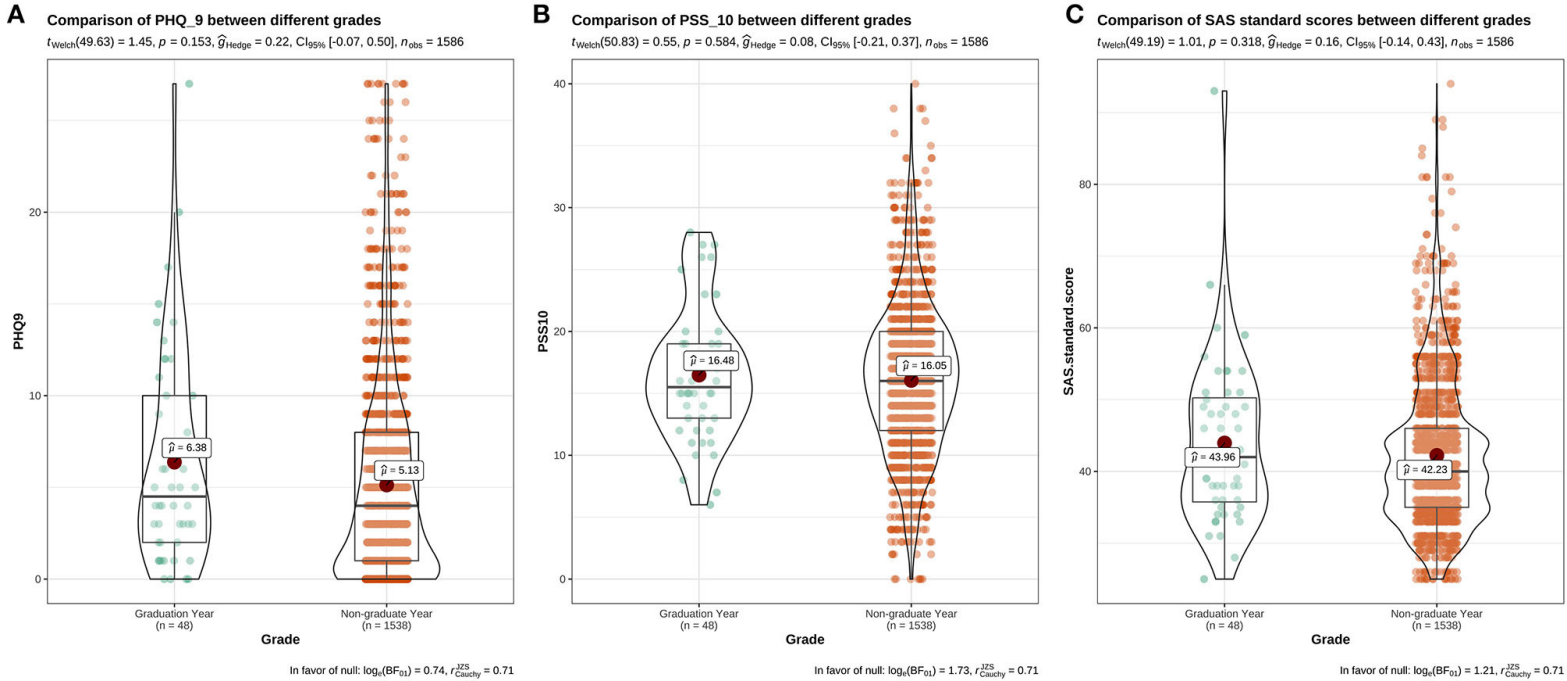
The distribution maps of PHQ-9, PSS-10, and SAS scores in different grades. **(A)** Distribution of PHQ-9 scores in different grades; **(B)** Distribution of PSS-10 scores in different grades; **(C)** Distribution of SAS scores in different grades.

**Figure 4:**
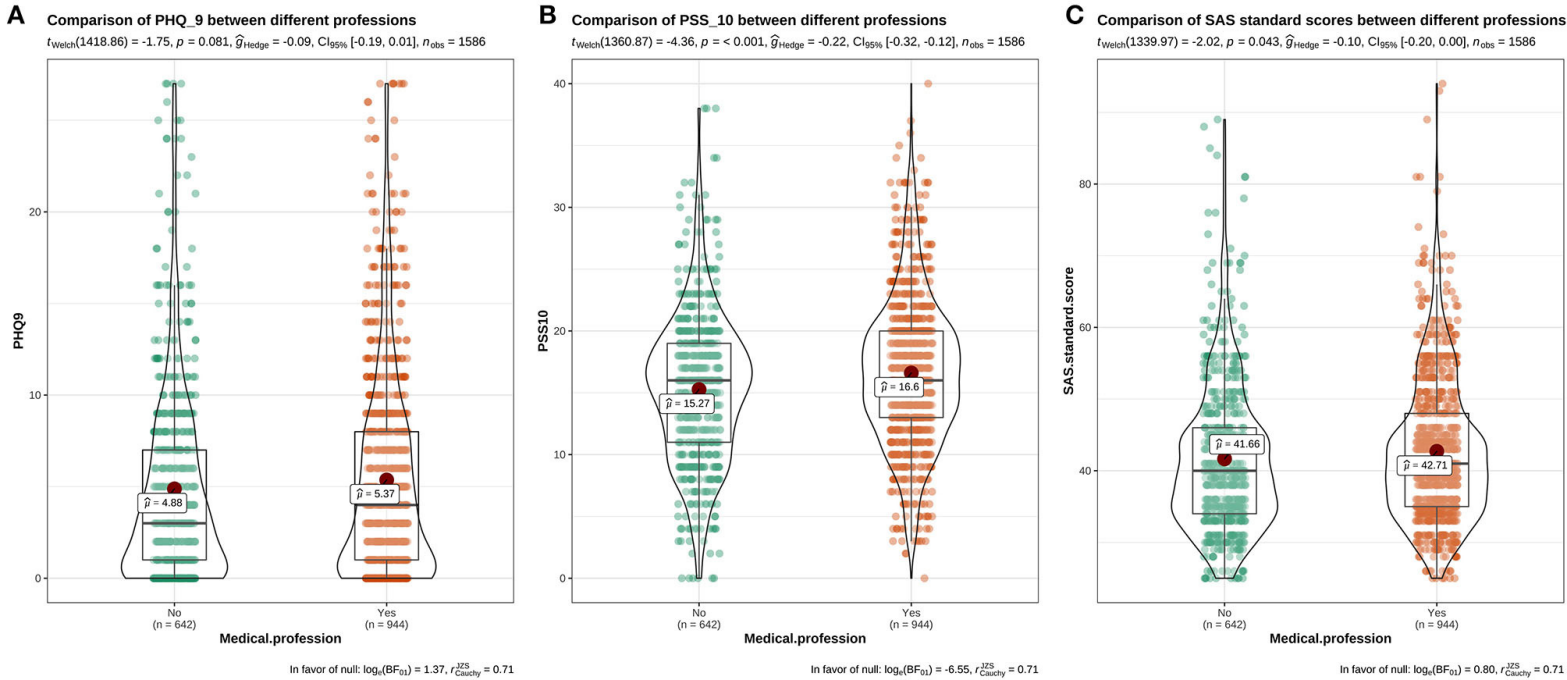
The distribution maps of PHQ-9, PSS-10, and SAS scores in different majors. **(A)** Distribution of PHQ-9 scores in different majors; **(B)** Distribution of PSS-10 scores in different majors; **(C)** Distribution of SAS scores in different majors.

**Figure 5:**
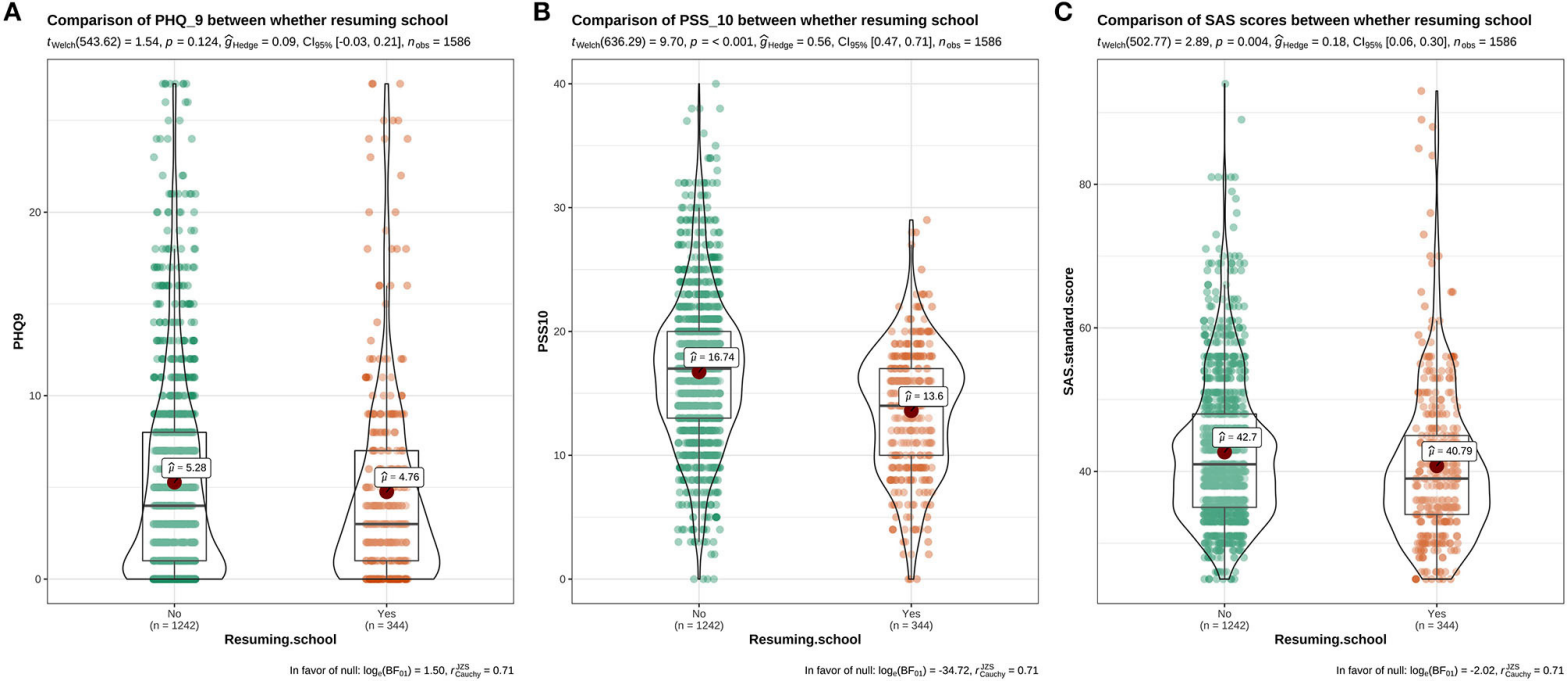
The distribution maps of PHQ-9, PSS-10, and SAS scores in resuming-school or not groups. **(A)** Distribution of PHQ-9 scores in the returned group and the non-returned group; **(B)** Distribution of PSS-10 scores in the returned group and the non-returned group; **(C)** Distribution of SAS scores in the returned group and the non-returned group.

The distribution of PSS-10, PHQ-9, and SAS of students with different genders, grades, and majors, who resumed school, and with different experiences of resuming school, can be seen in Figures 1–**5**. This chart mainly evaluated the distribution and difference of scores on PSS-10, PHQ-9, and SAS in different groups of college students. The greater the score, the more severe the degree of stress, depression, or anxiety. Taking Figure 1 as an example, we can clearly see the distribution of females and males in different degrees of depression, stress and anxiety. In the picture, the red dot represents the median: the median score in females was higher, demonstrating that females have higher levels of depression, stress, and anxiety. However, at the significant level of α = 0.05, there were significant differences between females and males in PSS-10 and SAS scores (*P* < 0.05); however, there was no significant difference in PHQ-9 scores (*P* > 0.05).

The results showed that there was a significant difference in the distribution of PHQ-9 scores among students with different psychological experiences (*P* < 0.05), indicating that there was a close relationship between depression and students with different psychological experiences. The negative experience of returning to school made students more likely to develop depression (**Figure 5**). There were significant differences in PSS-10 and SAS scores between male and female students. The median of stress (PSS-10) and anxiety (SAS) of female students was higher than that of male students (μ = 16.52>15.25, 42.7>41.55). It showed that the stress and anxiety levels of females are generally higher than that of males (Figure 1). There was no significant difference in stress perception, anxiety, and depression among different grades (*P* > 0.05) (Figure 2). Medical majors are significantly more sensitive to stress perception and more prone to anxiety than non-medical majors (Figure 3). There are also significant differences in stress and anxiety among college students who returned to school compared to those who did not (*P* < 0.05), and resuming school reduced students' stress and anxiety (Figure 4). Students with different psychological experiences (negative or positive) had significant differences in their perception of stress, anxiety, and depression (*P* < 0.05), and those with negative experiences had higher scores (Figure 5).

#### Principal Component Analysis of Different Populations

**Figure 6:**
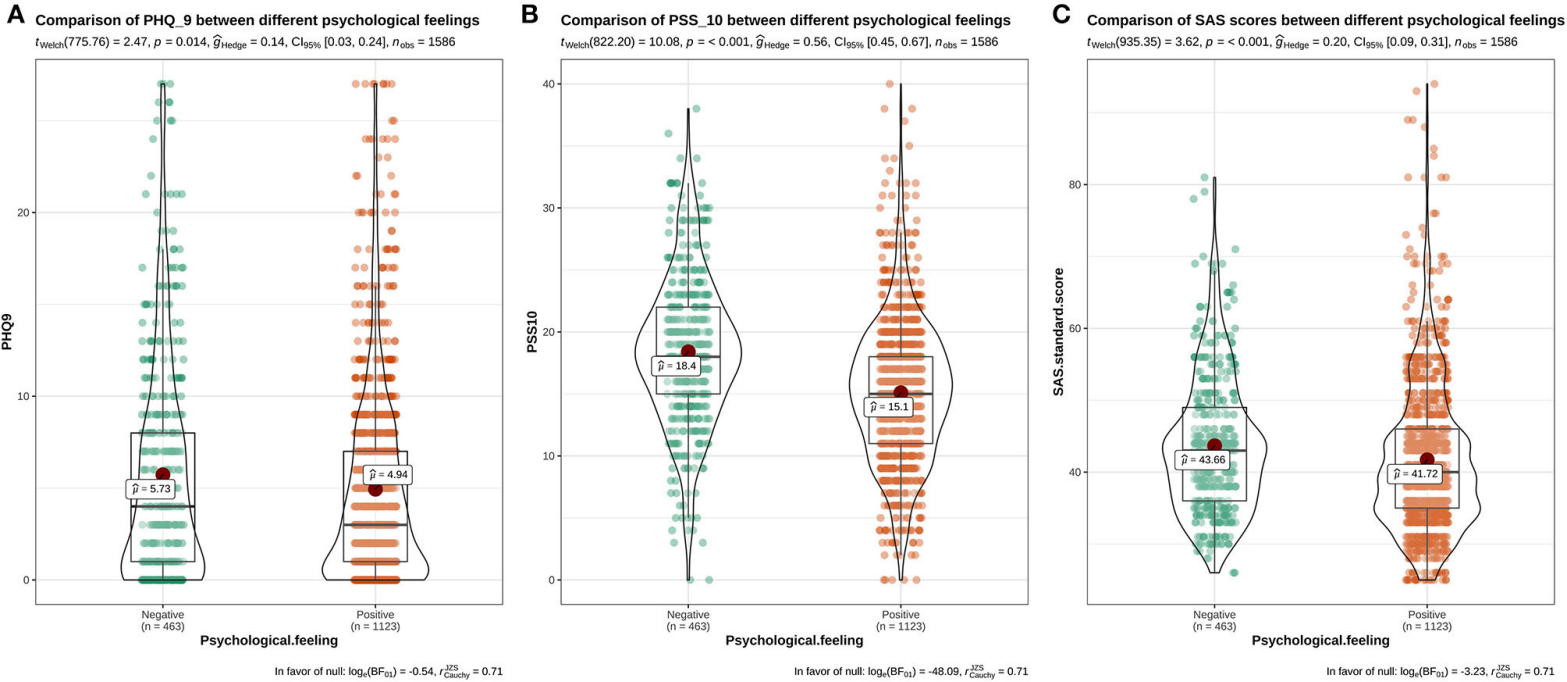
The distribution maps of PHQ-9, PSS-10, and SAS scores of students with different psychological experiences (negative and positive). **(A)** Distribution of PHQ-9 scores in different psychological experiences; **(B)** Distribution of PSS-10 scores in different psychological experiences; **(C)** Distribution of SAS scores in different psychological experiences.

In this study, principal component analysis was used to evaluate the correlation of PHQ-9, PSS-10, and SAS among different groups. The results showed that there were no significant differences among genders, grades, and majors. However, there was a relatively large difference between resuming and not resuming school (Figure 6). This demonstrated that resuming school can greatly relieve students' stress, anxiety, and depression.

## Discussion

The psychological quality and bearing capacity of college students is generally poor. The public health emergency caused by COVID-19, the sudden and public uncertainty it has caused, and its potential for serious harm are the main factors affecting college students' mental health. A study has shown that sudden public health events pose a challenge to psychological adaptability, especially in college students (12). As college students have less social experience and less knowledge about dealing with emergencies, fewer measures can be implemented in the face of critical situations in real life. Thus, when college students face public health emergencies of international concern, they may be at a loss as to what to do and extremely worried and afraid, which can easily lead to stress, depression, anxiety, and other emotions in the long-term. Notably, the delays in the recommencement of academic programs are positively correlated with anxiety symptoms (13). During the pandemic, the opening of colleges and universities was postponed in order to prevent the escalation of transmission. College students had to go out less, which may have resulted in their unwillingness to study and participate in social activities, further affecting their learning progress and employment difficulties, and aggravating their anxiety and depression. Although some colleges and universities recommenced in May 2020, some students who returned to school could not adapt to school life under the influence of the pandemic. Thus, it is worth noting that there are still problems in the mental health of college students, which cannot be ignored.

Growing evidence supports a clear association between COVID-19 pandemic and mental health. As we all know, people's mental health varies at different stages of pandemic development. A study has shown that the prevalence of possible psychopathological symptoms in China increased from the outbreak of COVID-19 to the remission period of COVID-19 (14). Our cross-sectional survey in May 2020 showed that college students had a relatively high detection rate of stress, anxiety and depression. This is closely related to the inevitable difficulties they faced during the investigation. On the one hand, with the gradual alleviation of the epidemic in China, college students across the country were faced with returning to school. Group meals, classes and bedtime may increase their fear of being infected. On the other hand, although the epidemic in China was gradually under control, the epidemic abroad was spreading rapidly. As foreign students, overseas Chinese and other overseas high-risk personnel had returned to China one after another, students were deeply worried about the uncertainty of the future development of the epidemic.

Under the influence of COVID-19, a higher proportion of people have a stress response (15). In response to an emergency, the body immediately mobilizes physical and psychological responses, called stress responses (16). Our study shows that the stress levels of college students was generally high before and after returning to school, and students with moderate stress levels or above (PSS-10 ≥ 14) account for 67.50% of the total, which is inconsistent with a previous study that showed that 28.79% of students had a stress response (17). The difference may be due to the fact that the inconsistency of the investigation date, we surveyed students from May 9 to 22,2020, while the study was conducted on February 19,2020. Moreover, the subjects in the previous studies were still staying at home at the time of study, while 20% of our subjects were already back at college. On one hand, with the extended time at home, the pressure on college students might also be gradually increasing. This may be due to their long-term lack of interpersonal communication, increased loneliness, inefficient online learning, imminent final exams, and other factors. On the other hand, the college students returning to school may have more pressure, because they are worried about being infected.

The detection rates of depression and anxiety among college students are relatively high (43.77 and 20.60%, respectively). This finding is slightly different from a previous study, which showed that the incidence of depression was 34.19% and that of anxiety was 21.34% (18). In that study, 76.8% of the students were non-medical majors, but in our study our subjects were all college students and nearly three-fifths (59.52%) were medical majors. Notably, a study confirmed that medical students scored higher in anxiety than non-medical students in terms of professional knowledge learning (19). Moreover, the incidence of depression was higher than that of anxiety in both of those studies. The higher risk of depressive symptoms may be related to the pandemic situation of COVID-19 (20). With their relatively developed online network, students can keep track of the development of the pandemic at any time, and will inevitably hear rumors. As a result, they are in a state of worry and fear every day, which aggravates their sense of depression.

Anxiety turned out to be the most prevalent and serious issue for female students. Table 1 shows that women account for nearly 2/3 of the subjects in our study. Therefore, the percentage gap between men and women may lead to high results of our research. Female are more likely to have excessive stress and anxiety than males, which is consistent with previous studies (15, 17, 21–23). Females are not only more emotionally sensitive than males, but are also more likely to think negatively, thus they are more likely to experience unhealthy psychological effects when facing a pandemic. The PHQ-9 scores of different genders had no statistically significant difference. Previous research has showed that the occurrence of depression in college students has nothing to do with gender (19, 24). However, the incidence of depression in different genders is not consistent with the results of some studies (3, 25). They believe that females are more likely to suffer from mild depression than males, perhaps because women are more sensitive and susceptible to tension. The reason for the inconsistency may be that the survey method used in this study is relatively simple, and the gender differences in depression need further investigation.

During the survey, only a small number of graduates accepted our questionnaire because of the great pressure of writing papers, looking for jobs and graduate re-examination. There was no significant difference in stress, anxiety, and depression between graduates and non-graduates, which may be due to the small sample size of 48 graduates (3.13%). But our results showed that graduates still had a slightly higher mental health impact than non-graduates. However, many studies have shown that graduates are more likely to have anxiety than non-graduates (17, 18, 23, 26, 27). During the pandemic, the postponement of school opening had a great impact on graduates. For example, medical graduates were unable to return to the internship unit to study clinical operations, which could affect their job prospects. In addition, graduates may face other issues such as pressure relating to writing their graduation theses, the pressure of employment, and the delay of re-examination for the postgraduate entrance examination, leading to an increase in the incidence of psychological anxiety (24).

Medical majors were more likely to experience excessive stress and anxiety than non-medical majors, which is a different result to that in previous research (3). The higher the awareness of COVID-19 among college students (both medical and non-medical students), the more accurately they will master prevention and control measures. At the same time, if they have a good ability to assess the validity of rumors, the incidence of stress reaction and anxiety will be reduced (28). However, less than half (43.7%) of the medical students had completely accurate knowledge regarding COVID-19 (23). Therefore, we speculate that there is no significant difference in the cognitive understanding of COVID-19 between medical majors and non-medical majors. This shows that the anxiety and stress faced by medical students may not be due to the influence of pandemic related factors. Chinese medical students face serious depression and anxiety because of difficulties such as the long time spent in school, academic pressure, pressure in clinical practice, and so on (19, 29, 30). Therefore, the stress experienced by medical students may come from the academic pressure associated with being a medical major. Compared with non-medical majors, medical students are more professional, study many subjects, and spend long in the academic system. With the delay in school commencement, they are more likely to experience stress, anxiety, and other emotions. In addition, most medical students practice in clinical hospitals. As they have just experienced the transformation from school to society, they have relatively insufficient clinical experience and a slightly poor mentality. When faced with the treatment of infected patients, they will feel extremely scared. Our research also shows that there is no significant difference in PHQ-9 among different majors. However, in terms of the incidence of depression in different professions (medical or non-medical), some previous studies had results that were the opposite of ours (19, 29, 30).

Compared with students who had returned to school, those who had not experienced significantly higher levels of stress and anxiety. This demonstrates that resuming school relieves students' sense of stress and anxiety to a certain extent. The negative emotions associated with resuming school (or news of resuming school) increase the likelihood that students will experience high stress levels and persistent anxiety. In addition, there are significant differences in PHQ-9 scores among students with different psychological experiences, and those with negative emotions are more likely to suffer from depression. In other words, negative reactions have a significant effect on student's mental health symptoms (31). Therefore, if we want to control the pandemic, we must maintain a positive attitude. Moreover, psychological counseling centers in universities should pay more attention to the psychological dynamics of student with negative emotions and implement accurate psychological intervention measures as soon as possible.

The results of principal component analysis showed that there were minimal differences among different genders, grades, and professional groups. On the contrary, the levels of stress, anxiety, and depression associated with the non-resumption of school were relatively high. In respect of the non-resumption of school (or late resumption of school), students face negative emotions brought about by suspension of academic activities (32). The negative effect of academic stress on students' anxiety and depression is significant (31). During the pandemic, learning changed from face-to-face teaching to online distant teaching; however, the low efficiency of online learning, prolonged learning time, long-term sitting, and dependence on mobile phones led to students' anxiety about learning results, self-negation and frustration (33). Ultimately, the pressure of learning increased, and caused anxiety about learning. Therefore, all relevant government departments and universities should pay close attention to the mental health status of students who have not resumed school (or have resumed school late), and implement psychological intervention (30). For example, colleges and universities can carry out activities “promoting peer encouragement and comfort” that impact on students' psychology (34). In addition, in order to regulate the negative physical emotions of college students, they can be encouraged to exercise properly (17), and student psychological counseling centers can be created (35). Universities need to focus on students with depression, which may manifest in cognitive impairment. Cognitive impairment refers to abnormalities in the brain's advanced intelligent processing related to learning, memory, and thinking judgment, resulting in serious learning and memory disorders. One study has suggested that cognitive impairment is the core symptom of depression (36). Therefore, for the groups with higher degrees of depression, it is particularly important to implement accurate psychological interventions.

In summary, during the pandemic, the psychological stress levels of college students are generally high, and there are still incidences of anxiety and depression. While the pandemic is still ongoing, college teachers should pay attention to the psychological status of students, especially female college students, who do not return to school (or return late), fresh graduates, and medical students. We believe that our study makes a significant contribution to the literature because it is the first study of its kind dealing specifically with the resumption of school. Relevant government departments and universities should strengthen psychological monitoring to manage college students and carry out extensive and in-depth health education and health promotion activities. In addition, our study does not involve the exploration of countermeasures, and effective intervention measures need to be further studied.

## Data Availability Statement

The raw data supporting the conclusions of this article will be made available by the authors, without undue reservation.

## Ethics Statement

The studies involving human participants were reviewed and approved by Academic Ethics Committee of Shaoxing People's Hospital. The patients/participants provided their written informed consent to participate in this study.

## Author Contributions

HZ designed the questionnaire. CZ participated in data collection. LZ carried out data analysis and visualization. HZ, CZ, MY, and XZ participated in the writing and revision of the article. XJ and LZ provided project funds. MY, LZ, and XJ are the authors of this article's juxtaposition newsletter. All authors contributed to the article and approved the submitted version.

## Conflict of Interest

The authors declare that the research was conducted in the absence of any commercial or financial relationships that could be construed as a potential conflict of interest.
